# Correlation between duration of untreated psychosis and long-term prognosis in chronic schizophrenia

**DOI:** 10.3389/fpsyt.2023.1112657

**Published:** 2023-02-16

**Authors:** Minglan Yu, Qingyu Tan, Yan Wang, Yi Xu, Tingting Wang, Dongmei Liu, Dechao Chen, Peiying Deng, Chaohua Huang, Xuemei Liang, Kezhi Liu, Bo Xiang

**Affiliations:** ^1^Department of Psychiatry, Laboratory of Neurological Diseases and Brain Function, Medical Laboratory Center, The Affiliated Hospital of Southwest Medical University, Luzhou, Sichuan, China; ^2^Department of Psychiatry, The First Affiliated Hospital, Zhejiang University School of Medicine, Hangzhou, Zhejiang, China; ^3^Department of Psychosomatic Medicine, People’s Hospital of Deyang City, Deyang, Sichuan, China; ^4^Department of Psychiatry, Yibin Fourth People’s Hospital, Yibin, Sichuan, China; ^5^Institute of Cardiovascular Research, Southwest Medical University, Luzhou, Sichuan, China; ^6^Central Nervous System Drug Key Laboratory of Sichuan Province, Luzhou, Sichuan, China

**Keywords:** duration of untreated psychosis (DUP), schizophrenia, cognition, clinical symptom, long-term prognosis

## Abstract

**Objective:**

To explore the relationship between the Duration of Untreated Psychosis (DUP) and long-term clinical outcome, cognitive and social function in patients with chronic schizophrenia (SCZ).

**Methods:**

A total of 248 subjects with chronic SCZ were enrolled in this study, including 156 in the short DUP group and 92 in the long DUP group. The Positive and Negative Symptoms Scale (PANSS), the Brief Negative Symptoms Scale (BNSS), the Global Assessment of Functioning (GAF) scale and the Repeatable Battery for the Assessment of Neuropsychological Status (RBANS) were used to assess all of the subjects.

**Results:**

The negative symptom scores (the PANSS and BNSS) of subjects with long DUP were significantly higher than that in subjects with short DUP. The scores of visual span and speech function in the short DUP group were significantly higher, indicative of decreasing cognitive function with time. In terms of social function, the short DUP group scored higher, with a statistically significant difference. Meanwhile, we found that the length of DUP was positively correlated with the negative symptom score of the PANSS, negatively correlated with visual span scores, and GAF scores.

**Conclusion:**

This study demonstrated that the DUP remained a significant association with negative symptom and cognition in long period of chronic SCZ.

## 1. Introduction

Schizophrenia (SCZ) is one of the most common and severe mental disorders, with an incidence of 1% (1). In China, SCZ is often stigmatized, which can lead to a longer period of mental illness without treatment (2). Duration of Untreated Psychosis (DUP) refers to the time interval between the first onset of psychotic symptoms and the first visit to the hospital and took antipsychotic drugs, and is a continuous psychiatric process (3, 4). The concept of DUP was introduced in recent years, and has since received a significant amount of attention as the length of DUP was related to treatment results and may have an impact on the prevention of SCZ (5). A large number of previous studies have suggested that longer DUP was associated with a poorer prognosis (6–8), including more severe clinical outcomes, decrease in cognitive and social function, some studies even believed that DUP can be used as an important predictor of prognosis (9–11). However, in these studies, the importance of DUP for prognosis is mainly confirmed in first-episode SCZ, whereas there are a few studies investigating the relationship of DUP with chronic SCZ. Therefore, this study discussed the relationship between DUP and long-term clinical outcomes in chronic SCZ.

Cognitive impairment is one of the core symptoms of SCZ, and it mainly manifests in memory, attention, processing speed, and executive function (12). The “neurotoxicity” hypothesis proposed by Wyatt et al. holds that the untreated period of mental illness is toxic to the brain, and such an effect could directly lead to cognitive impairment in patients with SCZ (13). The association between DUP and cognitive function has been discussed in some subsequent studies, but the results were not the consistent (14–16). Of course, these studies were based on first-episode SCZ.

To sum up, this study explored the relationship between DUP and long-term clinical prognosis, as well as cognitive and social function in patients with chronic SCZ, to provide a certain theoretical basis for future related research.

## 2. Materials and methods

### 2.1. Subjects

The participants with chronic SCZ were recruited at the Yibin Fourth People’s Hospital, PR China. The Structured Clinical Interview for Diagnostic and Statistical Manual of Mental Disorders, fourth edition (DSM-IV)-Patient Version (SCID-P) was used by two trained psychiatrists to interview and diagnose patients with SCZ (17). All patients were interviewed to assure if they had a history of psychiatric illness in their first-degree relatives. Inclusion criteria: (1) subjects who remained in long term hospitalization; (2) aged between 18 and 65; (3) minimum primary school education level; and (4) the total course of illness was 2 years or more. The exclusion criteria were organic mental illness, Parkinson’s, epilepsy, cerebral vascular disease, dementia, other serious physical illnesses, brain injuries, substance abuse or addiction, or inability to give informed consent. Finally, a total of 248 hospitalized subjects with chronic SCZ were enrolled in this study. The study was approved by the Medical Ethics Committee of the Fourth People’s Hospital of Yibin. All of the patients provided written informed consent for participation in the study.

### 2.2. Measures

In this study, we chose to use 6 months as the cut-off point, within 6 months for short DUP, and more than 6 months for long DUP (18, 19). Thus, of these 248 subjects, 156 were enrolled in the short DUP group and 92 in the long DUP group. At the same time, depending on the degree of cooperation of each subject, 71 subjects in the short DUP group and 33 in the long DUP group received cognitive and GAF tests. The assessment of DUP was obtained from family members and previous medical records in addition to semi-structured interviews with the subjects, so as to ensure the accuracy of the evaluation.

### 2.3. Clinical assessment

Two trained psychiatrists assessed all of the enrolled subjects utilizing the following scales: the Positive and Negative Symptoms Scale (PANSS) (20) and the Brief Negative Symptoms Scale (BNSS) (21) to evaluate the severity of symptoms, the Global Assessment of Functioning (GAF) Scale (22) to assess social function, and the Repeatable Battery for the Assessment of Neuropsychological Status (RBANS) (23) to evaluate cognitive function (immediate memory, visual span, verbal function, attention, delayed memory).

### 2.4. Statistical analysis

SPSS 22.0 was used for statistical analysis of the data. The Chi-square test and independent sample *t*-test were used for general baseline data and demographic characteristics. The independent sample *t*-test was used for the symptoms and cognitive and social function scores between the two groups. At the same time, to understand the correlation between DUP and symptom severity, cognitive and social function, DUP was logarithmically transformed to obtain approximately normal frequency distribution, and then Pearson correlation analysis was used. Data were expressed as mean ± standard deviation and *p* < 0.05 was considered statistically significant.

## 3. Results

### 3.1. Demographic characteristics

In our study, the median of DUP was 2.50 months (inter-quartile range, 1.00 to 14.50), the mean age was 41.01 ± 12.02 years, and the mean duration of follow up was 9.94 ± 9.12 years. A total of 113 of the patients were female (45.56%). The demographic characteristics are shown in Table 1. There were no statistically significant differences in gender, age, region, education, disease duration, suicide, smoking and drinking history between the two groups, but the familial heritability of the long DUP group was significantly higher than that of the short DUP group (χ^2^ = 5.269, *p* = 0.022) (Table 1).

**TABLE 1:** Demographic characteristics and baseline data.

	Short DUP group	Long DUP group	*t*/χ ^2^	*p*
Gender, *N*	156	92		
M (%)	81 (51.92)	54 (58.69)	1.070	0.301
F (%)	75 (48.08)	38 (41.31)		
Age	40.64 ± 11.94	41.65 ± 12.18	−0.639	0.523
Area (rural %)	117 (75.00)	63 (68.47)	1.237	0.266
Education years	8.21 ± 3.35	7.58 ± 3.83	1.366	0.173
Duration of disease	10.98 ± 9.32	11.92 ± 9.81	0.748	0.457
Family history (genetic %)	47 (30.12)	41 (44.56)	5.269	0.022
Prior suicide attempts (no %)	143 (91.66)	86 (93.47)	0.268	0.604
Do you drink alcohol (drinking %)	31 (19.87)	22 (23.91)	0.562	0.453
Smoking history (smoking %)	67 (42.94)	37 (40.21)	0.177	0.674
**Hospitalization**				
1-2 times (%)	40 (25.64)	23 (25.00)		
3-4 times (%)	77 (49.35)	45 (48.91)		
≥5 times (%)	39 (25.01)	24 (26.09)		
**The number and dose of antipsychotics**				
Clozapine (%)	38 (24.35)	13 (14.13)	6.023	0.177
Risperidone (%)	85 (54.48)	61 (66.30)		
Sulpiride (%)	9 (5.76)	8 (8.69)		
Aripiprazole (%)	16 (10.25)	6 (6.52)		
Quetiapine fumarate (%)	8 (5.16)	4 (4.36)		
Chlorpromazine equivalent	334.19 ± 134.42	359.78 ± 128.86	1.470	0.143

Data were presented as mean ± SEM (standard error of the mean). The difference of gender, area, family history, suicide, drinking habits, smoking history were analyzed by the χ^2^ test among two groups. Data of age, duration of disease, and education years were analyzed by the *t*-test. Short DUP, short duration of untreated psychosis; Long DUP, long duration of untreated psychosis; M, male; F, female. There was no statistical difference in the number of drugs used (χ^2^ = 6.023, *p* = 0.177). We converted the dose into the equivalent of chlorpromazine, and there was no difference between the two groups (*t* = 1.470, *p* = 0.143).

### 3.2. Medication and hospitalization

Table 1 shows the hospitalization of patients in both the groups; about 75% of the patients were hospitalized multiple times (Table 1).

In the two groups, we found the main antipsychotic drugs used included clozapine, risperidone, sulpiride, quetiapine fumarate, and aripiprazole tablets. We converted the dose of the above drugs into the equivalent of chlorpromazine, and we found that the dose difference between the two groups was not statistically significant (*t* = 1.470, *p* = 0.143) (Table 1).

### 3.3. Severity of symptoms

The scores of negative symptoms in the PANSS (*t* = –2.120, *p* = 0.046) and BNSS scale (*t* = –2.294, *p* = 0.023) in the long DUP group were significantly higher than in the short DUP group (Table 2). However, there was no significant differences between the positive, general and total PANSS scores (Table 2).

**TABLE 2:** Comparison of scale scores between two groups.

		Short DUP group (*N* = 156)	Long DUP group (*N* = 92)	*t*	*P*
The PANSS scale	Positive	24.31 ± 5.68	24.93 ± 6.61	−0.781	0.435
	Negative	18.74 ± 6.38	20.39 ± 6.03	−2.120	0.046^A^
	General	42.42 ± 9.13	43.82 ± 7.35	−1.253	0.211
	Total	85.48 ± 16.28	88.58 ± 12.98	−1.560	0.120
The BNSS scale		37.57 ± 14.33	42.16 ± 13.96	−2.294	0.023^A^

^A^The scores of the long DUP group was higher than that of the short DUP group, and the difference was statistically significant (*p* < 0.05).

### 3.4. Cognitive and social function

The scores of visual span (*t* = 2.155, *p* = 0.034) and speech function (*t* = 2.171, *p* = 0.032) in the short DUP group were higher than in the long DUP group (Table 3), but the remaining cognitive indicators showed no differences. In contrast, the short DUP group showed better social adaptability (*t* = –3.207, *p* = 0.002) (Table 3).

**TABLE 3:** Comparison of cognitive and social function.

	Short DUP group (*N* = 71)	Long DUP group (*N* = 33)	*t*	*P*
Immediate memory	21.43 ± 7.06	22.24 ± 9.18	−0.491	0.625
Visual span	30.70 ± 5.21	28.18 ± 6.23	2.155	0.034^A^
Verbal function	24.56 ± 3.92	22.30 ± 6.64	2.171	0.032^A^
Attention	41.84 ± 13.44	39.03 ± 16.73	0.918	0.361
Delayed memory	31.57 ± 9.13	30.27 ± 11.58	0.621	0.536
Total scores	149.91 ± 29.45	142.03 ± 42.00	1.104	0.272
GAF	61.57 ± 10.23	57.23 ± 10.41	−3.207	0.002^A^

^A^The scores of the short DUP group were significantly higher than that of the long DUP group, and the difference was statistically significant (*p* < 0.05).

### 3.5. Correlation analysis of DUP with symptoms, cognitive, and social function

**FIGURE 1:**
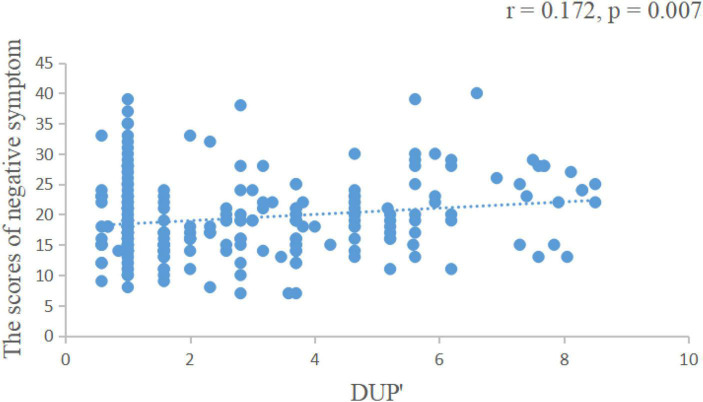
The length of DUP was positively correlated with the negative symptom score of the PANSS scale. DUP, duration of untreated psychosis; PANSS, positive and negative symptoms scale. DUP, DUP was logarithmically transformed to obtain approximately normal frequency distribution.

**FIGURE 2:**
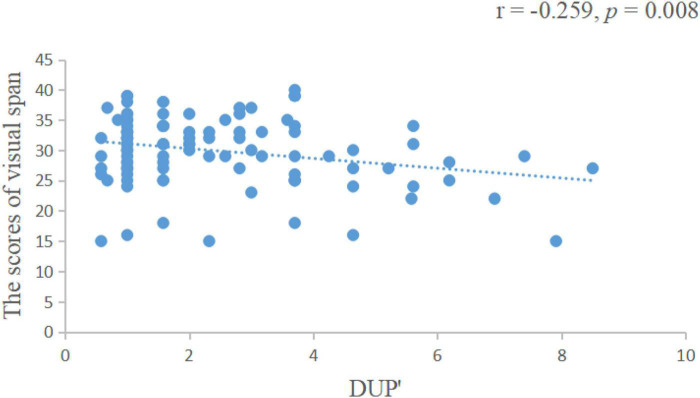
The length of DUP was negatively correlated with visual span scores. DUP, duration of untreated psychosis.

**FIGURE 3:**
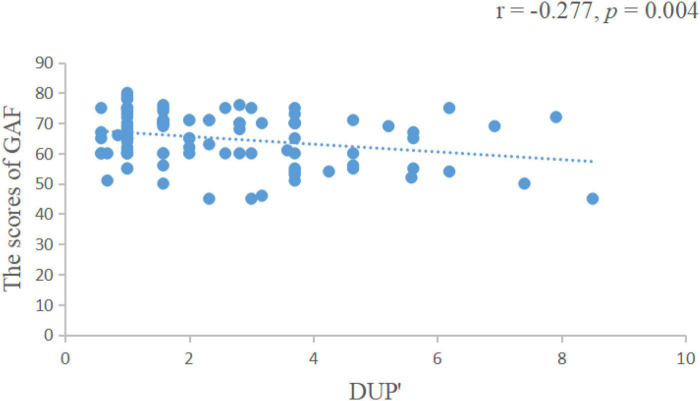
The length of DUP was negatively correlated with GAF scores. DUP, duration of untreated psychosis; GAF, global assessment of functioning.

We found that the length of DUP was positively correlated with the negative symptom scores of the PANSS scale (*r* = 0.172, *p* = 0.007, Figure 1), negatively correlated with visual span scores (*r* = −0.259, *p* = 0.008, Figure 2) and GAF scores (*r* = −0.277, *p* = 0.004, Figure 3).

## 4. Discussion

This study analyzed the relationship between DUP and long-term clinical outcome, as well as with the cognitive and social function of patients with chronic SCZ. We found that patients with untreated psychosis for longer than 6 months (long DUP group) demonstrated greater negative outcomes, including more severe negative symptoms, and worsened cognitive and social function than patients receiving treatment within 6 months of the onset of symptoms.

The DUP was defined in this study as the time interval between the first onset of psychotic symptoms and the first visit to the hospital. However, there was no consensus on the cut-off point for the length of DUP. A study by Cechnicki et al. found that the optimal cut-off point for DUP was week 23, beyond which a poor prognosis was more likely (18). At the same time, one study suggested that the prognosis would be better only when the DUP was less than 6 months (24), which was also applied to a previous study (25). The World Health Organization recommends that treatment for psychosis begin within 3 months of symptom onset, however, some studies have observed more than 3 months between the appearance of psychotic symptoms and treatment initiation in Chinese patients with SCZ (26, 27). Therefore, in accordance with previous studies, we chose to use 6 months as the cut-off point in the following analysis.

Previous studies have considered the DUP as one of the most common predictors of the outcome in SCZ, but mainly focused on the first-episode SCZ, and therefore its role in predicting long-term outcomes remains to be clarified (28). One follow-up investigation found that a longer DUP significantly predicted more serious clinical symptoms after 1 year (29). At the same time, a follow-up study by Cechnicki et al. also suggested that a longer DUP was positively correlated with a worse clinical outcomes (18), which was also supported by Ran mao-sheng et al. (19). In the present study, we found that DUP was positively correlated with the PANSS negative scale, and longer DUP with severe negative symptoms in schizophrenic patients treated for more than 2 years. Kanahara et al. identified that DUP was associated with greater negative symptoms and decreased functioning, but not with positive symptoms (27), at the same time, several studies also demonstrated that longer DUP hindered the improvement in PANSS negative, general subscales and total score in chronic SCZ (30–32). These findings also supported our results, especially showing increases in negative symptoms. Nonetheless, the cause of the association between DUP and negative symptoms has not been identified. Some researchers believed that there was an active and progressive pathological process prior to treatment, and if antipsychotic treatment delayed the structural changes in the brain associated with outcomes, the amount of neuronal damage would be related to the length of DUP, it was suggested that this neuronal damage hindered treatment response, leading to the greater residual negative symptoms in patients with longer DUP (12, 33). Meanwhile, other scholars suggested that, more serious negative symptoms as part of the underlying pattern of disease were one of the causes of prolonged DUP, and this type was associated with poor outcome (34, 35). This association is not yet clear and can be further explored in subsequent studies. Of course, long-term use of drugs in chronic SCZ may have an impact on the outcomes, but in our study, the results indicated that the length of DUP with more serious negative symptoms in chronic SCZ.

This study found that patients with longer DUP demonstrated worse cognitive and social functions. Goff et al. revealed that DUP can cause abnormalities in the hippocampus (36), and deep atrophy of the cortical sulcus, which have a critical effect on cognitive function (14). At the same time, some investigators suggested that the toxic effects of DUP may be biologically or psychologically mediated. It has been found that *N*-methyl-D-aspartic acid receptor hypofunction may cause glutamate excitotoxic damage in neurons (37). Meanwhile, the prolonged stress from untreated psychosis, can activate the hypothalamic–pituitary–adrenal axis, leading to greater glucocorticoid secretion, which can result in neuronal injury (38). These biological mechanisms, in turn, can lead to a worsened cognitive and social function in SCZ patients. It has also been suggested that there may be threshold effects in untreated psychiatric symptoms; thus, these neurotoxic effects can only be observed during a longer DUP (39). It was generally believed that the longer DUP, the greater cognitive deficits could be explained by these toxic effects (13). We also found that our results showed higher GAF scores than those of the Suffolk County study (11), one possible reason is that the patients with SCZ have been treated with medication in our study. However, some studies insisted that the DUP was unrelated to cognitive and social function (14, 40). The differences in these results may be explained by the method of evaluation, including collection of samples (first onset or chronic cases), study design, and statistical methods. Hence, these differences should be further evaluated.

The limitations of this study are as follows: First, the sources of measurement bias may have been present in our study, especially the key variable DUP. The assessment was obtained from patient interview, clinical case-notes and questioning of the relatives and carers, so as to reduce the recall bias and ensure the accuracy of the evaluation to the greatest extent. Meanwhile, in accordance with previous studies, we chose to use 6 months as the cut-off point, which would maximize the differences in outcomes between the two groups (18, 19, 32), therefore, follow-up studies should be carried out to determine a more appropriate cut-off point. Second, the sample size for the cognitive and GAF assessment was insufficient, and some findings did not pass multiple correction. Additionally, while examining cognitive and GAF function, the effect of patient’s psychopathology might override the impact of DUP. All of these have a certain impact on the results. Finally, when discussing the relationship between DUP and long-term clinical prognosis of chronic SCZ, other potential effects should be excluded, such as age of onset, premorbid personality, mode of onset, diagnostic subtyping, times of relapse and a more complex role of antipsychotic exposure.

## 5. Conclusion

The results of this study suggest that the DUP remained a significant association with negative symptom and cognition in long period of chronic SCZ.

## Data availability statement

The original contributions presented in this study are included in the article/supplementary material, further inquiries can be directed to the corresponding author.

## Ethics statement

The study was approved by the Medical Ethics Committee of the Fourth People’s Hospital of Yibin. The patients/participants provided their written informed consent to participate in this study.

## Author contributions

QT, MY, YW, and BX designed the experiment and wrote the manuscript. QT, TW, DL, DC, PD, CH, and XL collected and analyzed the data. KL and YX guided the manuscript writing. All authors have made significant scientific contributions to this manuscript and approved the final manuscript.
